# Bycatch in a bottle: what taxa are recoverable from metabarcoding DNA in historical invertebrate collection preservative fluid?

**DOI:** 10.1093/jhered/esag001

**Published:** 2026-01-12

**Authors:** Ajith Seresinghe, David Herbst, Jen Quick-Cleveland, Severyn Korneyev, Benjamin K Maples, Eva Sofia Horna Lowell, Malia Mosser, Robert N Fisher, Eric P Palkovacs, Daniel Gluesenkamp, Rachel S Meyer

**Affiliations:** Department of Ecology and Evolutionary Biology, University of California Santa Cruz, Santa Cruz, CA 95060, United States; Institute of Marine Sciences, University of California Santa Cruz, Santa Cruz, CA 95060, United States; Sierra Streams Institute, 117 New Mohawk Rd H, Nevada City, CA 95959, United States; Department of Ecology and Evolutionary Biology, University of California Santa Cruz, Santa Cruz, CA 95060, United States; California Department of Food and Agriculture, Plant Pest Diagnostics Branch, 3294 Meadowview Road, Sacramento, CA 95832-1448, United States; California Department of Food and Agriculture, Plant Pest Diagnostics Branch, 3294 Meadowview Road, Sacramento, CA 95832-1448, United States; San Diego Natural History Museum, 1788 El Prado Balboa Park, San Diego, CA 92101, United States; Department of Ecology and Evolutionary Biology, University of California Santa Cruz, Santa Cruz, CA 95060, United States; U.S. Geological Survey, Western Ecological Research Center, San Diego, CA 92101, United States; Department of Ecology and Evolutionary Biology, University of California Santa Cruz, Santa Cruz, CA 95060, United States; Fisheries Collaborative Program, Institute of Marine Sciences, University of California, Santa Cruz, CA 95060, Unites States; California Institute for Biodiversity, 1400 Shattuck Ave. STE #12 PMB 101, Berkeley, CA, 94709, United States; Department of Ecology and Evolutionary Biology, University of California Santa Cruz, Santa Cruz, CA 95060, United States

**Keywords:** benthic macroinvertebrate communities, eDNA, fungal associates, insect communities, natural history collections

## Abstract

Natural history museum collections are invaluable repositories of biodiversity, offering insights into life on Earth. Genomic approaches provide powerful tools to characterize biodiversity in these collections. However using these collections for genomics without damaging specimens is a challenge. Here, we develop and test non-destructive DNA metabarcoding methods to capture biodiversity from the preservative fluids of archived insect collections (‘*Bycatch’*). We optimized workflows for extracting and amplifying the partial *CO1* locus (*CO1*) and fungal *ITS1* locus from ethanol-based preservative fluids, validating ethanol preparation methods, comparing DNA extraction kits, and refining PCR protocols. Our results demonstrate that from museum collections with low DNA yields, *CO1* and *fungal ITS1* loci can often be recovered from preservative fluids, and we present detailed methodology and workflows. We test metabarcoding success to recover taxa in several museum collections ranging in age and storage condition. This is to support the State of California’s effort to catalog and sequence all insects and fungi, building baselines of California biodiversity with help from museum collections. Lastly, we investigate the complementarity of metabarcoding water versus ethanol and morphological identifications aimed to capture benthic macroinvertebrate biodiversity in streams. Our findings highlight that DNA metabarcoding of the preservative fluid is a non-destructive tool for capturing biodiversity in historical specimens, but there are limitations on the overlaps between DNA results and physical contents, where morphological identification still reigns in taxon counts, but metabarcoding sometimes provides more taxonomic resolution, and can be used to track DNA from other organisms such as fungi beyond the directly surveyed specimens.

## 1. Introduction

Recent studies have highlighted a global decline in insect diversity, abundance, and geographic range (Dirzo et al. 2014; Hallmann et al. 2017; Raven and Wagner 2021; Wagner et al. 2021). Addressing the decline of insect populations worldwide requires a multifaceted approach, but a critical first step is to thoroughly identify and describe insect species diversity. By systematically documenting and understanding existing diversity, researchers can lay the groundwork for effective conservation and management strategies to safeguard insect populations and the ecosystems they inhabit. Tapping into the rich resource of historical museum collections without sacrificing specimens is a scientific advancement that preserves voucher specimens and allows repeated sampling. This could potentially capture both historical and contemporary biodiversity data. Museum specimens serve as permanent reference material that allows researchers to physically verify molecular identifications, build reference libraries, and apply new taxonomic knowledge as it emerges. These collections provide leads and address knowledge gaps in insect biodiversity by building baselines for monitoring change over time.

High-throughput molecular-based methods such as DNA metabarcoding may offer a fast, efficient way to sample bulk archived insect collections. Metabarcoding may be particularly useful for characterizing a multispecies insect collection where sample sorting and morphological identifications have not been done or demand substantial time and technical expertise. Further, metabarcoding may help obtain more biodiversity associated with the collections than meets the eye or not. These collections are potential treasure troves of biodiversity information in thousands of museums and institutions that have remained largely unsequenced (Yeates et al. 2016).

Since microbial associates are likely present both within and outside of the insect specimens, metabarcoding insect preservative fluid can simultaneously capture associated fungi that was directly on the insects or was indirectly captured in the collections as fungi that occurred in the area where the insects were collected. Fungi, in particular, are well-documented associates of insects, often playing critical roles in their biology, such as in nutrition, defense, and even behavior (Moran and Jarvik 2010; Nicoletti and Becchimanzi 2022). Previous research has established that many insects harbor specific fungal symbionts (Suh et al. 2005). By targeting both insects and their fungi, we can inquire what the ecological relationships are among the insects, the fungi, and the provenance and timing of collections that help us gain understanding about the living world and what supports a community.

A traditional method of metabarcoding involves the sampling of bulk tissue, which can be destructive to the specimens contained in the samples. Non-destructive DNA metabarcoding of the preservative fluid has been proposed as an alternative method to characterize these collections (which we here term *bycatch*) (Carew et al. 2018; Martins et al. 2019; Zizka et al. 2019; Martoni et al. 2023). Non-destructive metabarcoding involves just the sampling of the DNA present in the preservative fluid, maintaining the physical integrity of the specimens. Bulk insect samples are often stored in a preservative fluid such as ethanol. Bulk samples often used in bycatch metabarcoding studies are most commonly clean-picked, i.e. separated from sediment, plant material, or any other organic material in the sample and placed in clean ethanol. However, many collections that reside in museums may not have been clean-picked. The presence of this ‘dirty’ material in original bulk samples is thought to introduce contaminants, PCR inhibitors, and non-target organisms that can bias the detection of the invertebrate community present in the sample. Additionally, quality and purity of the preservative fluid along with varying storage conditions create uncertainties in whether the DNA present in the fluid will remain intact for metabarcoding. For example, light exposure, temperature, and time itself may degrade DNA into smaller fragments (Wandeler et al. 2007). Furthermore, most of the studies that have used non-destructive metabarcoding leverage more contemporary collections with well-known preservation history, but plenty of collections have unknown histories.

In this study, we aim to test whether metabarcoding of the preservative fluid (DNA bycatch) suffices as a high-throughput method to sample mixed natural history collections. We define the DNA present in the preservative solution outside of target specimens as *Bycatch*. There is interest from California-wide bioinventory initiatives to sequence all insects and fungi in tandem and to leverage environmental DNA techniques to complement physical collection surveys. We first use fresh benthic macroinvertebrate (BMI) samples from two stream sites to test how metabarcoding taxonomic profiles vary based on different DNA extraction methods. We compare three commercial extraction kits in capturing arthropod and fungal diversity in these two samples. We use these results to select and optimize methods to sample a variety of natural history collections from across California and assess the quality of taxonomic inventories using metabarcoding. These samples range in age (fresh to decades old), condition, and preservation history. We then use the data generated from those initial two benthic macroinvertebrate samples to comprehensively compare the taxonomic profiles generated from the ethanol samples that were metabarcoded to stream water eDNA samples that were metabarcoded and to morphological identifications of hand-sorted collections. In this comparison, we ask what biodiversity in water and in ethanol preservatives is missing in the identified BMI collections, and conversely, what biodiversity is missed by molecular methods. Our overarching goal is to inform the California-wide initiative All Taxa Bioinventory (ATBI), a voucher-based DNA-powered inventory of California biodiversity with some insight into how beneficial or cumbersome metabarcoding data from bottle bycatch can be.

We developed workflows centered around three main aims:

(1) Determine if contemporary invertebrate collections stored in ethanol contain ‘bycatch DNA’ in the preservative fluid suitable for metabarcoding, and select an extraction kit that is suitable for retrieving arthropod and fungal DNA from only 5 mL of ethanol.
(2) Assess how differences in sample collections (e.g. storage conditions, sample type, media, age) affect observed *CO1* arthropod community profiles.
(3) Compare morphological and molecular taxonomic identifications (from both ethanol preservative and water eDNA) using two kicknet aquatic survey samples.

## 2. Methods and materials

### 2.1 Study sites & design

**Fig. 1:**
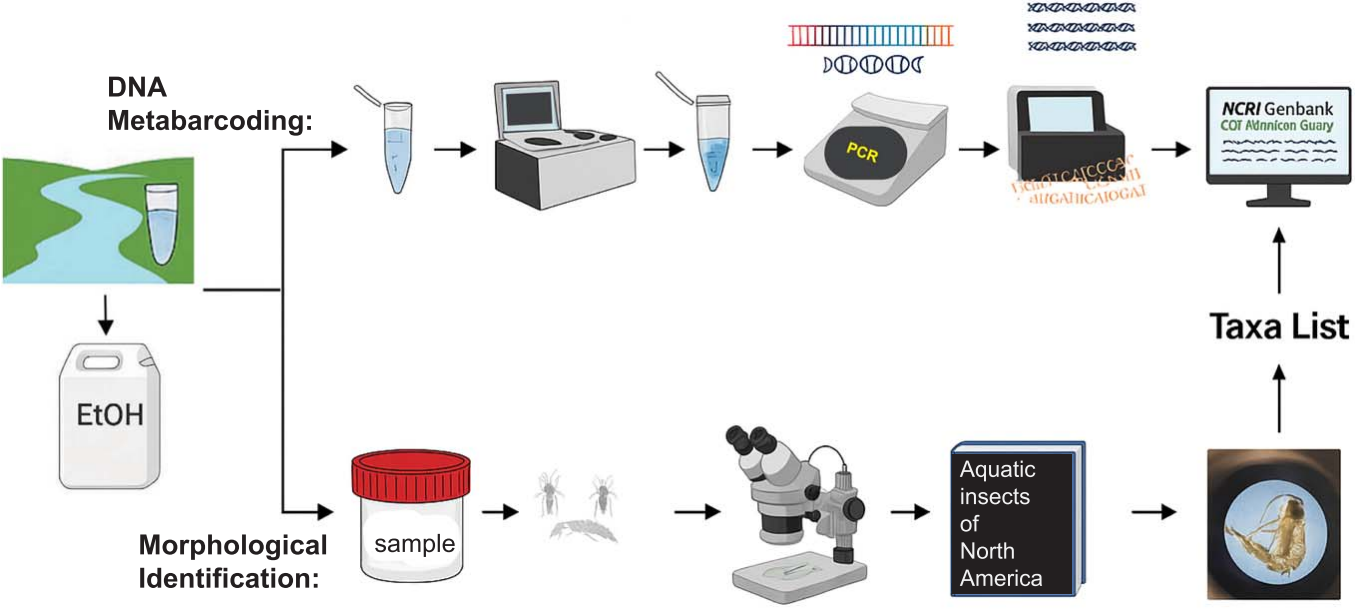
Workflow illustrating two parallel approaches for generating benthic macroinvertebrate taxa lists from ethanol-preserved bulk samples collected at the Lower Yuba River and Deer Creek 10. The top path depicts the molecular workflow, including DNA extraction, PCR amplification, sequencing, and taxonomic assignment via BLAST against a reference database. The bottom path outlines the morphological workflow, where samples are subsampled, organisms are sorted and identified to the highest possible taxonomic resolution using morphological keys. Both workflows result in taxon lists that can be directly compared to assess the complementarity of DNA metabarcoding and traditional morphological identification.

Study sites were located in the Sierra Nevada mountain range of California (Supplemental Table S3). Aquatic kicknet surveys were conducted between June 2022 and October 2022. Samples from two sites within the Yuba River watershed were used for this study: the Lower Yuba River (hereafter, LYR) and its tributary Lower Deer Creek (hereafter DC10). The invertebrate samples from these two sites were used to compare and optimize different methods for extracting DNA and to validate molecular techniques against traditional morphological analyses (Fig. 1).

### 2.2 Methods for BMI sample collection

We conducted aquatic kicknet surveys using a 30-cm wide D-frame net with a 500-μm mesh to collect invertebrates. For each of 11 locations representing mixed habitat of riffle and pool, the substrate within a 30 cm x 30 cm area immediately upstream of the net was disturbed, and invertebrates, along with organic material, were collected. After pooling samples from each transect in a bucket, large debris (sticks, leaves, rocks) was removed. The sample was then strained through a 100-μm mesh to remove excess water and transferred into a 500 mL polypropylene container with 95% ethanol for preservation. Samples were stored at 4°C until processing. Subsequently, the pooled benthic sample was split using a Folsom plankton splitter to obtain a minimum count of 550 organisms. Organisms were sorted to the highest taxonomic resolution possible, generally to genus or species level, except for oligochaetes, ostracods, and flatworms. These identifications were assigned using the latest keys and literature (e.g. Merritt et al., 2019). Organisms were then counted, and densities were calculated for each site based on the total area sampled.

Stream water eDNA (water eDNA hereafter) samples for metabarcoding were collected from the LYR and DC10, where benthic macroinvertebrate surveys were also conducted. At each site, stream water was filtered through 0.45 μm Millipore Sterivex Pressure filters, then the excess water was pressed through the filter, and 1 mL Zymo RNA/DNA Shield (R1100–50) was injected into the filter with a 3 mL syringe and sealed with Luer locks. Filters were frozen at −20°C until DNA extraction.

*For ethanol samples:* We used a SpeedVac (Thermofisher Scientific, Waltham, MA, USA) to remove ethanol and ready samples for DNA extraction by concentrating the DNA and tissue while simultaneously eliminating the ethanol or other fixative. We limited ethanol draws to 5 mL to consider that some specimens do not have much fixative and this volume is what museum curators were comfortable allowing to be sacrificed. To start the SpeedVac evaporation, the bulk sample in preservative fluid was vortexed for at least 10 s. Next, because a typical SpeedVac does not accept tubes of 5 mL volume, we pipetted 1.67 mL of the ethanol into each of three low-bind microcentrifuge tubes. To each tube we added 2 μL of 3 M sodium acetate and briefly vortexed them. Sodium acetate is used to facilitate the precipitation of the free DNA contained in the sample. We placed tubes into a SpeedVac set to high heat and let the liquid evaporate most of the way (~200–300 μL). We pooled the contents of all three tubes into a single low-bind microcentrifuge tube, being careful to retrieve all material. We concentrated the pooled sample tube again by SpeedVac until only a pellet remained. We resuspended each pellet with 800 μL of CD1 (lysis buffer) and vortexed the mixture for at least 10 s. From here, the Qiagen DNEasy PowerSoil Pro kit (Cat # 47016; Qiagen Inc., Germantown MD, USA) was followed to extract DNA per the manufacturer’s instructions (Starting at step 2). We included an extraction blank for every round of samples extracted. All samples were eluted in 70 μL.

The Qiagen DNEasy PowerSoil Pro kit was chosen to use after vetting it against two other kits. The results of those tests are detailed in our results under Sections 3.2 & 3.2. The other two kits were the Qiagen DNeasy Blood and Tissue kit and the Magbio Highprep Blood and Tissue Kit (Cat # 69506; Qiagen Inc., Germantown MD, USA; Cat # HPBTS-D96; Magbio Genomics Inc., Gaithersburg, MD, United States). For both of those kits, the same methods were used where the SpeedVac method was employed and pellets were resuspended in the respective lysis buffer for each kit. The kit protocols were closely followed in subsequent steps to extract DNA per the manufacturer’s instructions. We used three extraction replicates per site and kit but lost one extraction replicate for DC10 extracted with the Powersoil Pro extraction kit.

### 2.3 For water filters

Water filters were lysed in 900 μL of buffer ATL tissue lysis buffer (Qiagen Inc., Germantown MD, USA) and 100 μL of 20 mg/mL proteinase K (Omega Biotek, Norcross, GA, C753P75) for 24 h in a shaking incubator at 54°C shaken at 130 rpm. A combination of filter scraps and lysate was the input material for Qiagen Powersoil Pro Kit protocol, skipping the mechanical lysis and starting from the humic acid precipitation step (step 5).

### 2.4 Library preparation

The NGS library preparation methods following the DNA extraction methods were closely followed as described by Ruiz-Ramos et al. (2023). All DNA extracts were quantified using a Qubit High Sensitivity kit (Invitrogen, Carlsbad, CA, USA) on a Varioskan Lux plate reader. We chose to use the partial *CO1* region [mlCOIintF, GGWACWGGWTGAACWGTWTAYCCYCC (Leray et al. 2013) and Fol-degen-rev, TANACYTCNGGRTGNCCRAARAAYCA (Yu et al. 2012)] to target and amplify a 333 bp fragment of invertebrate DNA. PCR cycling conditions for *CO1* were as follows: initial denaturation at 95°C for 5 min, followed by 13 touchdown cycles (94°C for 30s, annealing starting at 69.5°C for 90s decreasing by 1.5°C per cycle, and 72°C for 90s), then 35 cycles of amplification (94°C for 30s, 50°C for 30 s, and 72°C for 60s), with a final extension at 72°C for 10 min. The *ITS1* region, which here we called FITS [ITS5, GGAAGTAAAAGTCGTAACAAGG (White et al. 1990) and 5.8S_fungi, CAAGAGATCCGTTGTTGAAAGTT (Epp et al. 2012)], was used to target and amplify a 350 average bp fragment of fungal DNA. FITS amplification conditions were: initial denaturation at 95°C for 5 min, followed by 13 touchdown cycles (94°C for 30s, annealing starting at 69.5°C for 30s decreasing by 1.5°C per cycle, and 72°C for 60s), then 35 cycles of amplification (94°C for 30s, 50°C for 30s, and 72°C for 60s), with a final extension at 72°C for 10 min. We ran three half reaction 15 μL PCRs for each marker on each sample using 2 μL of template, 7.5 μL of Qiagen Multiplex PCR Plus Kit mix (Qiagen Inc. Germantown MD, USA), 0.15 μL of 10 uM forward primer, and 0.15 μL of 10 uM reverse primer. This amounted to three PCR replicates per marker for every sample. Replicates for each marker were checked on a 2% agarose gel to visualize and confirm DNA amplification. PCR replicates were pooled and cleaned with MagBio HighPrep PCR Beads at a ratio of 1.2 to 1. The cleaned product was quantified using a Qubit Broad Range kit (Invitrogen, Carlsbad, CA, USA) on a Varioskan Lux plate reader. Post quantification, equimolar quantities of both markers were pooled by sample. About 2 μL of pooled amplicons were then dual indexed using Nextera XT Index Plate A (Illumina, San Diego, CA, USA). Indexing reactions were performed in 25 μL and contained 0.625 μL of each index, 12.5 μL of Kapa Hifi HotStart Ready Mix (Kapa Biosystems, Woburn, MA, USA) with the following PCR conditions: hold at 95°C for 5 min, then 12 cycles of 20 s at 98°C, 30 s at 56°C, and 3 min at 72°C, followed by a final extension at 72°C for 5 min, and 7°C hold. Indexed amplicons were checked on a 2% gel to confirm the success of the indexing reaction and cleaned again with beads at a 1.2 to 1 ratio. Libraries were quantified again with the Qubit Broad Range kit as above and were pooled at equimolar quantities for a final library concentration of ~ 50 nM. This was sequenced on a Illumina MiSeq platform with a 600 cycle kit aiming for 50,000 paired reads per marker and sample at the Paleogenomics Sequencing Facility at UC Santa Cruz.

For analysis on historic museum samples, metabarcoding library preparation closely followed the methods described previously with the following alterations described below. Once we extracted and quantified samples using the methods described above, we found that many of the historic samples had extremely low yields. For this reason, PCR was performed with 7 μL input and increasing cycles for *CO1* to accommodate the low yields. We also changed our bead ratio for our second clean-up because an update to MiSeq kit chemistry made the machine more sensitive to adapter dimers so we were stricter to remove small fragments with a 1:1 PCR product to bead ratio. The final library was then sequenced at the Paleogenomics Sequencing Facility at UC Santa Cruz on an Illumina Nextseq 2000 platform with a cycle kit aiming for 100,000 paired reads per marker and sample.

### 2.5 Sequence processing

The sequence processing methods for our extraction kit test closely followed the methods described in Ruiz-Ramos et al. (2023). The Anacapa Toolkit (Curd et al. 2019) and CRUX reference databases were used to filter reads by quality, sort the reads, and assign taxonomy to amplicon sequence variants. The read aligner program Bowtie2 was then used to align ASVs to reference databases and make taxonomic calls (Langmead and Salzberg 2012). The Bayesian Lowest Common Ancestor algorithm was employed to make sure taxonomic classification summaries were at least to 70% Bayesian Confidence Cutoff, following Curd et al. (2019). Taxonomy tables that had taxonomic classification summaries of 95% Bayesian Confidence Cutoff were chosen and used for final analysis. These taxonomy tables were then input to MS Excel and decontaminated by removing singletons and taxa that had more reads in extraction and PCR blanks. For the *CO1* taxonomy tables, we filtered taxa to just arthropods for our extraction kit test. For *FITS* we used all taxa on the taxonomy tables for our comparison.

In preparation for the remaining metabarcoding work, we chose to compare two taxonomic assignment workflows: Anacapa Toolkit (Curd et al. 2019) and Tronko (Pipes and Nielsen 2024), which both rely on reference databases built from NCBI Genbank (no data only in BOLD was in the reference databases). Anacapa uses Bowtie2 for accurate alignment to a custom database and probabilistic accuracy (BLCA) for assignments. Tronko uses a phylogenetic approach for mapping where the ASV is placed on a database of phylogenetic trees made from reference sequences. We ran Tronko with a more aggressive LCA cutoff of 5 that assigns taxa by aggregating all reference tree nodes with alignment scores within 5 units of the best-scoring node which will be more likely to keep smaller clades that enhance resolution. The Tronko pipeline was chosen for downstream analyses due to its higher taxonomic resolution and recovery of more taxa (Table S1). Taxonomy tables for *CO1* & *FITS* were decontaminated in Rstudio using the decontam package with the prevalence filter setting set to 0.1 (Davis et al. 2018; R Development Core Team, 2024). Taxa with only one sequence read across all sample libraries were removed.

Fastq files were made publicly available on NCBI Bioproject PRJNA1405772, Amplicon Sequence Variants and their taxonomic determinations were deposited in Zenodo under https://doi.org/10.5281/zenodo.18336436, and reference databases are available in Zenodo under https://doi.org/10.5281/zenodo.10049246.

### 2.6 Data analysis

To select extraction kits for metabarcoding invertebrates and fungi, we compared profiles of arthropod and fungal taxa in metabarcoding results from the different extraction methods, and selected methods with the greatest number of recovered arthropods and fungi. We also examined which of the recovered genera were also found in morphological identifications (see Section 2.2 for morphological methods) and factored the total of these in our decision of which DNA extraction method to proceed with. Results are displayed as heatmaps of arthropods recovered in metabarcoding taxonomy tables, and these were made with Ranacapa (Kandlikar et al. 2018).

To compare recovery of benthic macroinvertebrate biodiversity in riverine ecosystems, we compared morphological and molecular taxonomic identifications from kicknet aquatic survey samples at LYR and Deer Creek (DC10). Additionally, we incorporated water eDNA metabarcoding from these sites to evaluate the concordance of these three survey methods. The molecular data were pruned to retain only freshwater Arthropoda, Annelida, Mollusca, Platyhelminthes, as these groups were the primary biological targets of BMI surveys, and the taxa hand-picked, sorted, and identified from the bulk samples collected. We also kept the groups Cnidaria, and Porifera since they may be of interest as non-target taxa. In the molecular dataset, sequence reads for all retained taxa were pooled across biological replicates. This approach was applied only to the non-destructive metabarcoding methods, as there were no biological extraction replicates for the water eDNA data. To ensure consistency in taxonomy, Taxize (Chamberlain and Szöcs 2013) was used to query given morphological ID names to the NCBI taxonomic backbone, from the most resolved classification, to ensure the morphological and molecular data were comparable.

To compare molecular and morphological data at LYR and DC10, we estimated taxonomic richness, tallied concordant taxa, and tallied taxa more resolved in each method. We first assessed how taxonomic diversity from metabarcoding compared to morphological taxonomic diversity without manipulating the taxonomic outputs such as combining taxon lineages of different ranks (which we do in the second assessment). In this comparison, all unique taxon lineages in the molecular dataset, including those with BOLD accession numbers, were retained. Each taxon lineage was categorized at the species, genus, family, order, or class level, and the total number of assignments were calculated for each site and metabarcoding method. Morphological identifications of benthic macroinvertebrates were totaled across all taxonomic ranks, allowing a direct comparison of taxonomic assignments from each method. For our second assessment, we compared metabarcoding results to morphological identifications by restricting molecular data to taxonomic assignments that aligned with the morphological data structure of SAFIT Level Two Taxonomic Resolution (SAFIT, Richards and Rogers, 2011), which specifies genus or species-level identification for all groups where possible, and is a standard level of identification in BMI biomonitoring (Mazor et al. 2016). SAFIT Level Two is useful because it is used in regulatory assessments such as the California Stream Condition Index score CSCI (Mazor et al. 2016). We provide details on this framework in the Section 2.7 and include the scoring system in resulting supplemental tables.

### 2.7 Framework for SAFIT level two comparison scoring

If there was concordance for a genus for both the morphological and metabarcoding data, and if there was a species detected within that genus as well, we allowed the species and the genus to be included for the comparison. In metabarcoding data, If the species-level taxon name was a genus name followed by a BOLD accession number, the genus-level assignment was used for comparison. Additionally, if a morphological identification was assigned to subgenus and the corresponding metabarcoding assignment was at the genus level, the subgenus was considered to provide higher taxonomic resolution.

## 3. Results

### 3.1 Arthropod recovery is consistent across kits

Using kicknet samples, we tested differences in DNA recovered from samples extracted with three different kits, starting with Speedvac concentration of 5 mL ethanol: Qiagen Blood & Tissue (hereafter QBT), Magbio Blood & Tissue (hereafter MBT), & Qiagen PowerSoil Pro kit (hereafter PS). We found DNA yields varied substantially & we saw no significant relationship between DNA yields and PCR band intensity (Table S3.3).

**Fig. 2:**
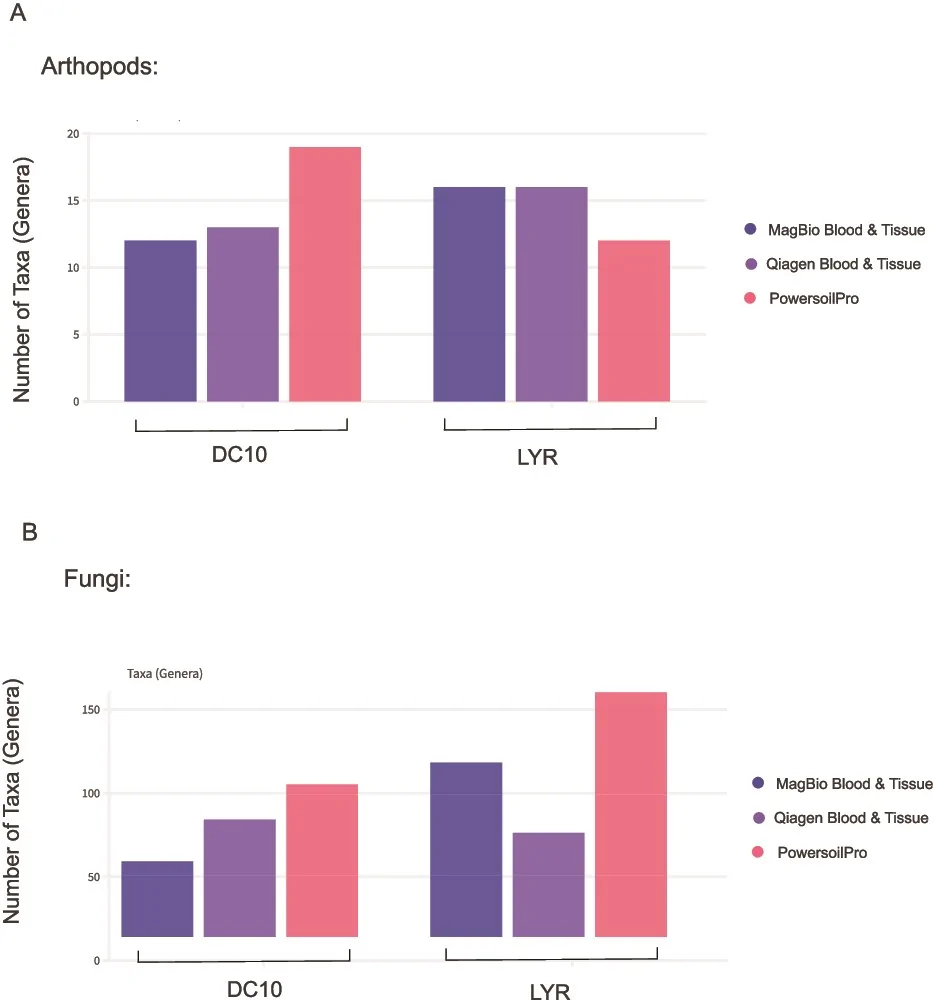
(A) Bar graph of arthropod directions by BMI sample from the three different extraction kits. Y-axis indicates the number of genera detected in each BMI sample by each extraction kit. BMI sample name under grouped bars. Across both samples there was no substantial difference in the number of arthropod genera captured by each extraction kit. (B) Bar graph of fungi detections by BMI sample from the three different extraction kits. Y-axis indicates the number of genera detected in each BMI sample by each extraction kit. BMI sample name under grouped bars. In both samples, the PowerSoil kit captured many more genera than both blood and tissue extraction kits.

**Fig. 3:**
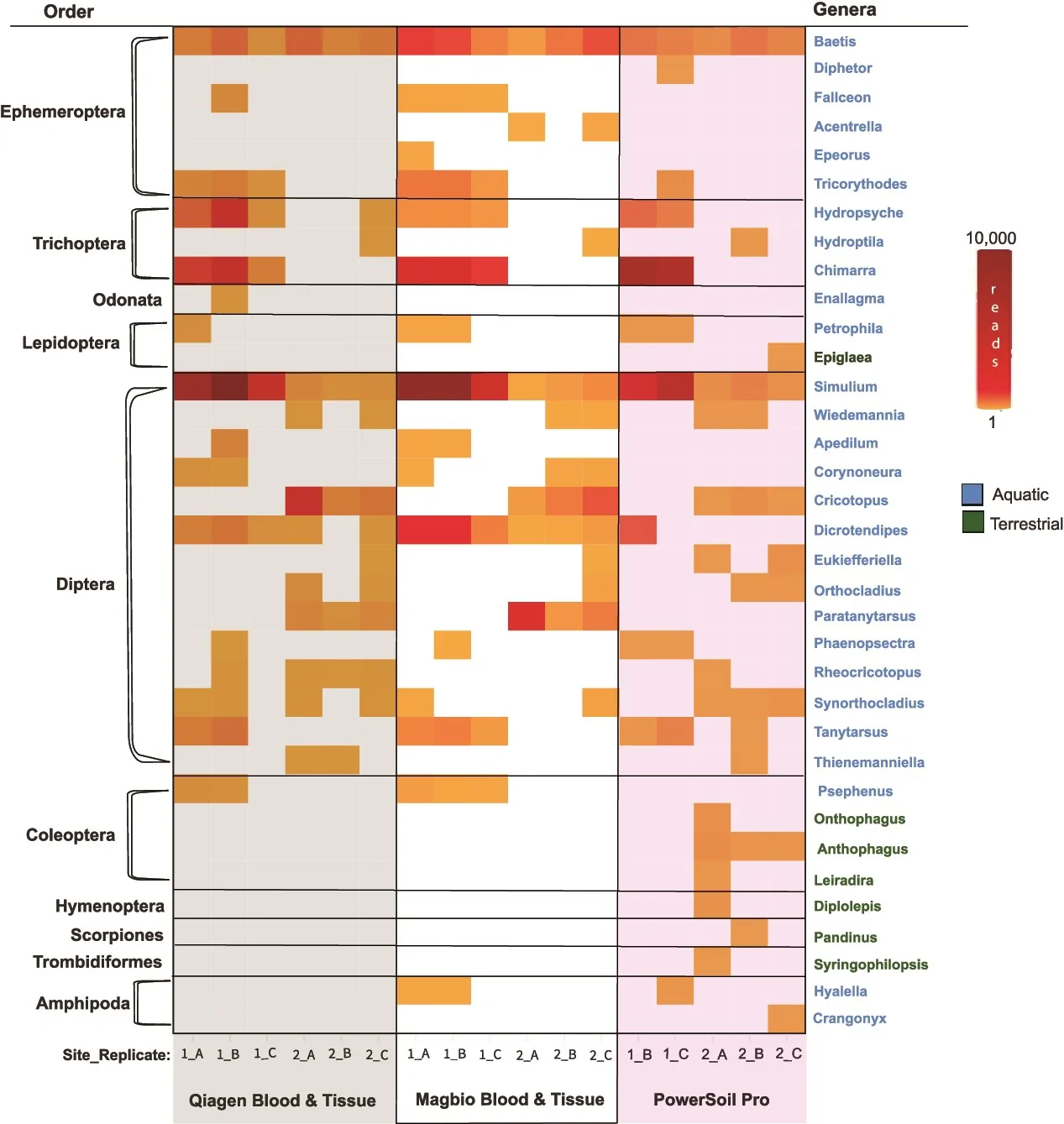
Heatmap of arthropod genera read abundance in different DNA extracts from three kits and two BMI samples. Within sample names, ‘1’ refers to sample ‘Deer Creek 10’ and 2 refers to ‘Lower Yuba River’. Within sample names, biological replicates are shown as A, B, and C. We lost one replicate for PowerSoil Pro ‘Deer Creek 10’. White color is absence.

All extraction kits recovered similar numbers of assigned taxa for each sample, with the PS kit capturing the highest diversity of genera at DC10, and both Blood and Tissue kits capturing the highest diversity at LYR (Fig. 2A; Table S2). In terms of arthropod composition, we found both Blood and Tissue kits were more similar in arthropod composition than the PS kit was to either of them (Fig. 3). The PS kit demonstrated better capture of terrestrial arthropods. We found between technical replicates we could not capture the community composition consistently. When taxa were shared among all three kits, we saw similar relative abundance of reads (Fig. 3).

In assessing arthropod taxa validated by morpho-IDs, both Blood and Tissue kits recovered more genera than the PS kit, but the difference was small, with only a couple of taxa unique to each extraction kit (Table S2). Morpho-ID taxa that were more abundant by physical counts of individuals were more consistently captured across kits and technical replicates. For example, the black fly larvae of *Simulium* and the common mayfly *Baetis* (Fig. 3) were two of the most abundant Morpho-ID taxa and were consistently abundant in all metabarcoding results (Table S2; Fig. 3). However, some morpho-ID taxa were often present in only one extraction replicate, and some were consistently missed by all kits. We concluded there were no clear gains in arthropod data by extraction kit for small ethanol sample bycatch metabarcoding.

### 3.2 PowerSoil pro kit enhances fungal detection

In the kicknet sample comparison of DNA extraction kits, the PS kit captured ~ 2.5-fold more fungal classes that were not detected by the Blood and Tissue kits. For example, when compared to the MBT kit, the PS kit recovered 16 overlapping classes, while detecting 11 additional classes of fungi not captured by the MBT kit (27 total). At more resolved taxonomic levels such as genus level, the PS kit captured a much broader array of fungal taxa compared to both blood and tissue kits (Fig. 2B). Many taxa were missed by both Blood and Tissue kits (628 lineages found in PS versus 365 fungal lineages in MagBio Blood & Tissue and 378 lineages in Qiagen Blood & Tissue). We selected the PS kit for downstream analyses because we observed clear gains in fungal taxa and no loss in arthropod information.

### 3.3 Arthropod recovery from museum samples varies with sample age and processing method

**Fig. 4:**
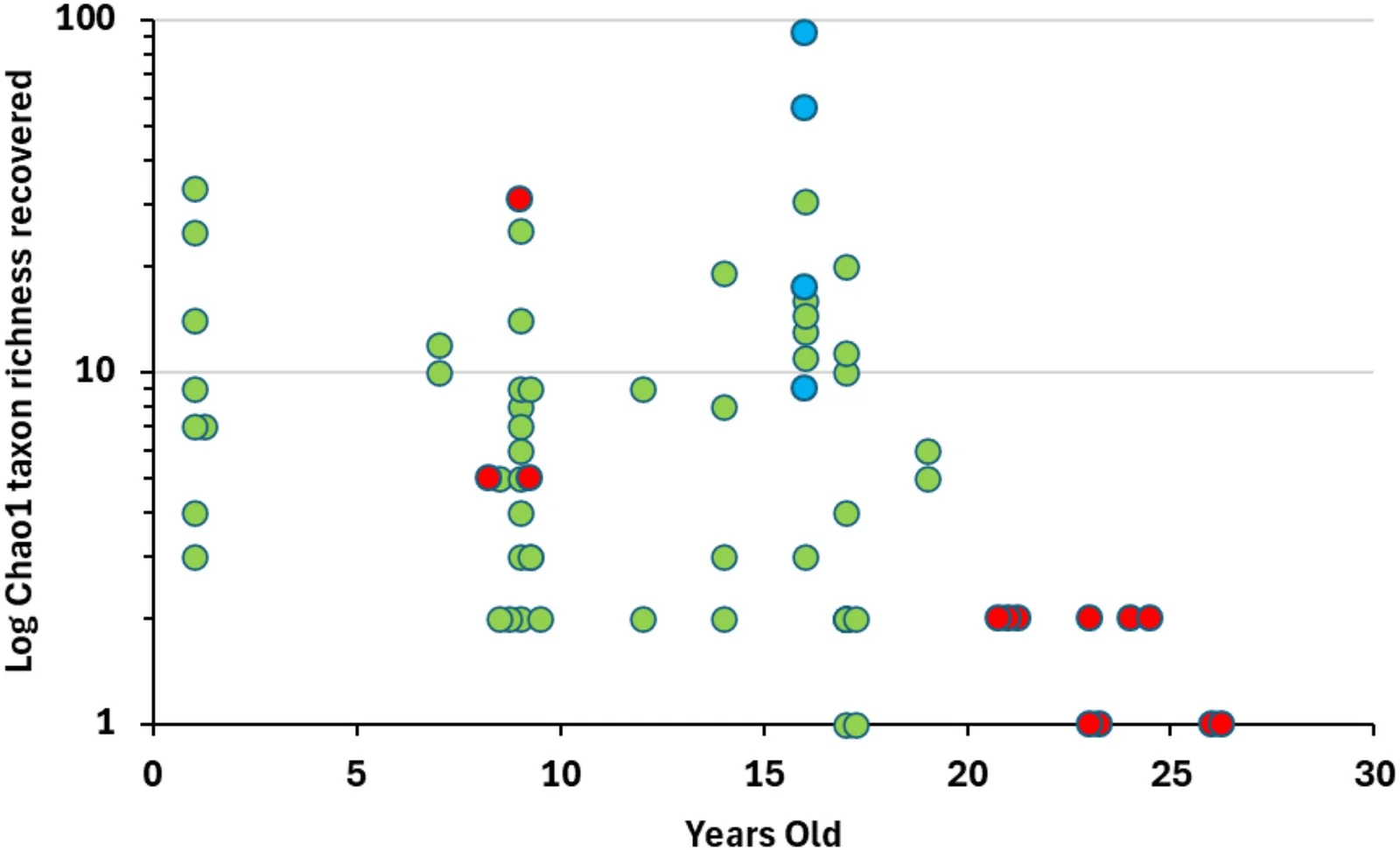
Scatter plot showing log Chao1 richness estimates for museum samples across different sample ages. Data was filtered to just arthropod taxa. Colors represent green for malaise traps and red for pitfall traps and blue for the samples that come from a single particular site that was very diverse. The plot suggests that there is variation in richness owing to time and sample source, with older samples often yielding fewer arthropod taxa, indicating possible aging or collection method effects on DNA preservation and recovery from museum samples. The data include both filtered and unfiltered subsampling.

Our museum collections (Table S2.1) that represented a variety of sample types and storage conditions exhibited varying success in arthropod recovery, with sample age being a significant factor on DNA recovery and arthropod richness. Across all museum samples (*n* = 70), we found a significant relationship between sample age and the alpha diversity metric Chao1 (KW, *P* = 0.00025). Older samples generally yielded fewer arthropod taxa (Fig. 4), but some samples up to 20 years old did exhibit high arthropod richness. We were unable to recover any arthropod taxa from the oldest collection samples from the USGS WERC collection (1998–2003). However, this batch of samples were from pitfall traps and also contained the smallest ethanol volumes, with many samples < 1 mL, limiting our ability to perform extractions under standard conditions, so age alone could not be disambiguated from fixative volume used as the DNA source. Interestingly, we did see a band in gel electrophoresis of the *CO1* PCR product for these samples, but the sequencing results had many reads of human DNA (Table S1.5), with no arthropod taxa detected.

Paired two-tailed T-tests (Table S3.2) showed that samples processed via direct pipetting ethanol contained significantly higher arthropod richness than those processed using 190-micron filtration of that ethanol prior to DNA extraction, as indicated by the Observed richness (*P* = 0.021), Chao1 (*P* = 0.011), and Se.Chao1 (*P* = 0.008) indices. In contrast, there were no significant differences between the two methods for diversity indices that account for evenness, such as Shannon (*P* = 0.644) and Simpson (*P* = 0.759) (Table S3.2). This suggests that while the pipetting method recovers more arthropod taxa overall, the relative read abundance of taxa remains similar between the two approaches. We also found no significant effects on community composition linked to sampling method (PERMANOVA, *P* = 0.884, supplemental Fig. S1), suggesting while the pipetting method recovered more taxa, the overall compositional profile of the arthropod community across samples is similar.

### 3.4 Fungal community composition and insect-associated taxa in historic museum collections

The PS kit DNA from all museum collections produced > 1589 diverse taxonomic lineages from the FITS metabarcode, largely of Ascomycota and Basidiomycota (Table S1.6). Kicknet PS samples produced 628 lineages, 354 overlapping with the museum collections.

The museum invertebrate samples displayed a variety of fungal taxa representing the fungal biome on invertebrate bodies and museum storage, though it is possible these taxa also reflect the ecology of the sample provenance as well. Samples that produced no invertebrate metabarcodes still yielded fungal data. The most prevalent families are Trichosporonaceae (in 70/70 of samples), Cladosporiaceae (53/70 samples), Malasseziaceae (50/70 samples), Aspergillaceae—(35/70 samples), and Cordycipitaceae (25/70 samples).

*Fungal biome of invertebrate bodies:* Among the prevalent families detected, Cordycipitaceae stood out because many of its members are entomopathogens (insect pathogens), providing one of the clearest indicators of true insect-associated fungi in our museum samples (Bu et al. 2025). *Beauveria* (Cordycipitaceae) are an entomopathogenic group of fungi such as B. bassiana, found in 19 samples, and the recently described *B. pseudobassiana* (7 samples), which was only formally separated from *B. bassiana* in 2011 based on molecular data (Rehner et al. 2011). Remarkably, three samples collected in 2008 (B1, B15, and B32) contained *B. pseudobassiana* DNA, 3 years before this species was even formally described (Rehner et al. 2011). This detection of *B. pseudobassiana* in museum collections predating its formal description highlights how molecular methods can reveal cryptic diversity that morphological identification would have missed. Likely these collections would have also been identified as *B. bassiana* at the time of collection. Some individual samples harbored up to four *Beauveria* species simultaneously, revealing complex pathogen communities preserved in decades-old malaise trap collections. Metschnikowiaceae (142 643 reads), a common family, contains *Metschnikowia* yeasts, found in 16 samples, included *Microstictia pimensis* and *M. chrysoperlae*, both named for the insects from which they were first isolated (Suh et al. 2005). These fungi taxa are important symbionts of these insects and are associated with their digestive tracts (Yoshihashi and Degawa 2023). Nectar and flower-associated yeasts were particularly well-represented, including *Starmerella* (12/70 samples) which are closely tied to bees and even a part of their microbiome.

*Fungi attributed to the museum and human handling artifacts:* Trichosporonaceae are globally distributed and occupy both free-living saprophytic niches (e.g. soils, decomposing wood, water, and food substrates) and host-associated niches on humans and animals, reflecting broad ecological flexibility that likely contributes to their ubiquitous detection across all museum ethanol samples (Aliyu et al. 2020). *Cutaneotrichosporon* (Trichosporonaceae) was highly prevalent across samples (68/70), and because members of this genus are predominantly associated with human or animal hosts rather than insects (Aliyu et al. 2020), its ubiquity in museum ethanol likely reflects human or storage-related contamination rather than insect-associated fungi. Cladosporiaceae being common among samples is consistent with members of this family, such as Cladosporium (53/70), being among the most ubiquitous environmental and indoor molds (Bensch et al. 2012), suggesting their presence reflects general storage-related contamination. Malasseziaceae were frequently detected in museum samples, which is unsurprising considering they are obligate animal and human skin yeast (Gaitanis et al. 2012). Their presence in the preservative ethanol is most likely attributed to human handling rather than with the insects themselves. Aspergillaceae (e.g., Aspergillus) are typical indoor and storage molds (Mousavi et al. 2016), so their presence in museum ethanol reflects storage contamination.

### 3.5 Application to riverine ecosystems: Comparing morphological and molecular data

**Fig. 5:**
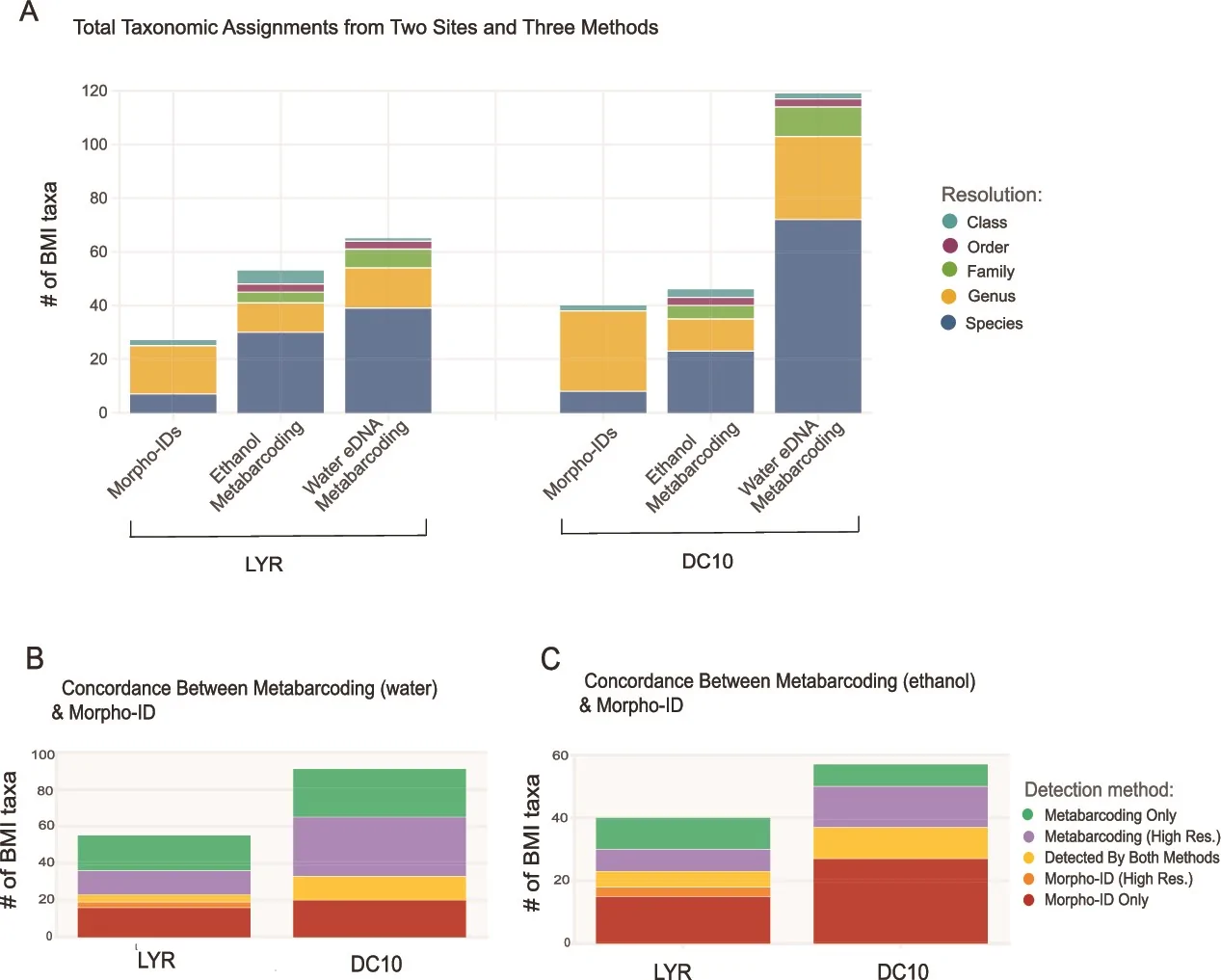
Taxonomic assignment and concordance assessment. (A) Stacked bar plot showing taxonomic assignments across the three different methods: Morphological identifications (morpho-IDs), non-destructive metabarcoding (ethanol metabarcoding), and water eDNA metabarcoding at the Lower Yuba River (LYR) and Deer Creek 10 (DC10). Bars represent the number of taxa assigned at each taxonomic level. The bar plots show how molecular methods, particularly water eDNA capture greater species level assignments while morphological and molecular approaches capture similar level of genus level assignments. (B) Stacked bar plot depicting the concordance between non-destructive metabarcoding of preservative fluid (ethanol metabarcoding) and morphological identifications (morpho-IDs) for each sample. The color scheme represents different detection patterns: Red indicates taxa detected uniquely by morpho-IDs, orange denotes taxa resolved to higher taxonomic levels by morpho-IDs, yellow represents taxa with a perfect match between both methods, purple highlights taxa identified at higher taxonomic resolutions by metabarcoding, and green indicates taxa detected only by metabarcoding. The plot demonstrates a substantial number of taxa detected solely by morpho-IDs, while metabarcoding captures some unique taxa and many resolved to finer taxonomic levels, illustrating the complementarity of these methods. (C) Stacked bar plot depicting the concordance between water eDNA metabarcoding and morpho-IDs for each sample. The bar plot demonstrates the robust signal of BMI biodiversity produced by water eDNA, with many detections unique to water eDNA and many taxa resolved to higher levels.

Framework 1:

In our first comparison, we found that metabarcoding methods captured greater species-level assignments than traditional morphological identifications (Fig. 5A). This pattern was particularly pronounced in the water eDNA dataset that yielded the highest number of unique species-level assignments at both the LYR (39 species) and DC10 (72 species). Ethanol metabarcoding also recovered more species assignments than morpho-IDs but to a lesser extent with 30 species detected at the Lower Yuba River and 23 species at DC10. In contrast to morpho-ID taxa which identified only seven and eight species at the two sites the LYR and DC10 respectively. At the genus level, however, morpho-IDs and metabarcoding methods captured similar levels of variation. At DC10 genus-level assignments were nearly identical between morphological identifications and water eDNA metabarcoding while ethanol metabarcoding recovered 12 genera. At the LYR, a similar trend was observed where morphological identifications recovered 18 genera, while water eDNA and ethanol metabarcoding detected 15 and 11 genera, respectively. Water eDNA metabarcoding consistently captured the highest diversity, followed by ethanol metabarcoding, with morphological identifications recovering the least variation. This pattern was most striking at the species level where water eDNA captured most variation in comparison to both ethanol metabarcoding and morpho-ID taxa.

Framework 2:

For the second comparison, we directly compared metabarcoding results to morphological identifications by restricting molecular data to taxonomic assignments that aligned with the morphological data structure of SAFIT Level Two Taxonomic Resolution (Fig. 5B). We first compared our ethanol metabarcoding to morphological identifications. At the LYR, morpho-IDs detected 15 taxa not detected by metabarcoding while metabarcoding only detected 10 taxa not detected by morpho-ID. Five taxa were detected by both methods. Metabarcoding provided greater taxonomic resolution for seven taxa, whereas morpho-IDs resolved three taxa to finer levels. In DC10, morpho-IDs identification uniquely detected 27 taxa, while metabarcoding identified seven unique taxa. Ten taxa were detected between both methods. Metabarcoding improved resolution for 13 taxa, while no taxa were resolved to finer levels through morpho-IDs. For both sites, metabarcoding missed many taxa identified by morpho-IDs, but also captured a substantial number of taxa missed by morpho-IDs. For genera found in both methods, metabarcoding assigned more of these to species level than morpho-ID did (Fig. 5B).

The metabarcoding results for water samples produced more taxa than metabarcoding from ethanol. At the LYR there were 16 taxa uniquely detected by morpho-IDs and 19 detected only by metabarcoding, with four taxa in common (Fig. 5B). Metabarcoding assigned species for 13 genera, compared to three cases where morpho-IDs provided higher resolution identification. At DC10, water eDNA metabarcoding detected 26 unique taxa, while morpho-IDs detected 20, with 13 taxa in common. Metabarcoding provided finer resolution for 32 genera, while no taxa were identified to a higher resolution by morphology.

Water eDNA metabarcoding identified the greatest number of unique taxa, while ethanol metabarcoding detected fewer (Fig. 5B). Overall, there was greater concordance between water eDNA metabarcoding and morpho-IDs compared to ethanol metabarcoding and morpho-IDs. Morpho-IDs captured many unique taxa that were not detected by either metabarcoding method. However, when taxa were detected by both approaches, metabarcoding often provided higher taxonomic resolution.

### 3.6 Combining morphological and molecular approaches yields most complete stream diversity profiles

While traditional kicknet surveys captured key Ephemeroptera, Plecoptera, and Trichoptera (EPT) taxa, both water and ethanol metabarcoding missed several of these, including the stoneflies at each site respectively: *Calineuria californica* and *Hesperoperla pacifica* at DC10, and *Skwala* at the Lower Yuba. Conversely, metabarcoding provided finer resolution of taxonomic groups that are often under-resolved in morphology-based assessments. For instance, aquatic oligochaetes are typically left at the subclass level in our morpho-IDs (Oligochaeta), but both water and ethanol metabarcoding assigned many of these to genus and species levels, expanding our taxonomic resolution. In particular, the water eDNA samples from both DC10 and LYR yielded diverse and well-resolved oligochaete profiles.

Sequence read counts did not correlate with physical organism counts. Our side-by-side comparisons showed no consistent relationship between read profiles and morphological abundance data (data not shown). As such, using sequence reads as proxies for relative abundance risks producing misleading results about how the aquatic community is structured.

Metabarcoding also revealed misidentifications and cryptic biodiversity. At both sites, an invasive amphipod, *Crangonyx floridanus*, was detected via both ethanol and water eDNA, despite being initially misidentified as *Gammarus* in morphological assessments. Upon closer inspection, these specimens were subsequently reidentified and confirmed as *C. floridanus* through morphological verification. This example highlights the ability of DNA metabarcoding to flag specimens for reexamination and uncover hidden diversity, offering valuable leads for re-curating collections and species lists.

In another instance, water eDNA from the LYR detected the invasive freshwater jellyfish *Craspedacusta sowerbyi*. While unlikely to be living in the stream, this taxon may originate from upstream reservoirs. Manual BLAST verification supported its identity, and GBIF records confirm its presence in nearby Sierra Nevada water bodies. This illustrates how water eDNA can track biodiversity signals from upstream sources, providing early warnings for species introductions and insights into regional freshwater connectivity. These tools are critical to freshwater ecosystem management in a rapidly changing landscape.

## 4. Discussion

In this study, we aimed (1) determine whether DNA metabarcoding of preservative fluids could serve as a non-destructive method for characterizing museum insect collections and identify optimal extraction methods, (2) assess how collection characteristics affect DNA recovery from historical specimens, and (3) compare the biodiversity captured by morphological identification versus molecular approaches in freshwater systems.

Our non-destructive extractions of the preservative ethanol served as a high-throughput method to sample bulk invertebrate collections for DNA metabarcoding. By using a non-invasive approach, we were able to preserve specimen integrity. This was critical for our study as the specimens in some of our bulk collections were subsequently used for both morphological identifications and single specimen barcoding. The Speedvac method was efficient for processing smaller ethanol volumes (0.5–5 mL) but may become impractical for larger volumes. Handling greater amounts of ethanol requires multiple microcentrifuge tubes, increasing sample transfers and risking the loss of small DNA pellets during pooling. This quickly becomes time-consuming and inefficient as sample ethanol volume increases. To address these challenges, previous studies have employed filtration-based methods, such as passing preservative ethanol through a 0.45 μm filter (Zizka et al. 2019).

### 4.1 Arthropod detection is inconsistent across DNA extraction replicates

Detection of arthropod taxa varied across extraction replicates, with some taxa verified by morphology appearing in only one replicate. However, the overall community compositions of each extraction replicate were similar (Fig. 3). This contrasts with other studies; for example, Carew et al. (2018) and Martins et al. (2019) found that extraction replicates of the same sample reliably yielded consistent taxa and community profiles. Martins et al used ethanol from bulk samples that were collected in the field only days to weeks prior, and that never had an ethanol change, which is completely different from how museum specimens are (where you have multiple, often uncounted solute changes, and specimens are many years old). Some studies apply mild lysis treatments to enhance DNA release before ethanol subsampling (Batovska et al. 2021; Miraldo et al. 2025) in a way that does not impede specimen morphological identification, but the studies rely on fresh (<1 year old) collections. Martoni et al. (2019), for example, found that pre-lysing specimens and using the lysis in non-destructive extractions not only yielded *COI* barcodes but also improved morphological identification of Diptera larvae. We suspect there are a few contributors to the low replicability we encountered: (1) we did not homogenize subsamples but took separate ethanol draws that are expected to be somewhat heterogeneous, (2) no pre-treatments were used, and (3) the composition of samples included rare taxa (in biomass) where vortexing and pipetting step could have caused varying quantities of body parts and small organisms to be suspended in solution, leading to heterogeneity in each subsample.

Despite the presence of specimens determined by morpho-IDs in the physical samples, DNA metabarcoding of sample bycatch in ethanol often failed to consistently detect the full diversity of the collection in each extraction replicate. Even with pooling the detections of each extraction replicates for the LYR and DC10, there were still substantial discrepancies between morpho-IDs and ethanol metabarcoding. In both the Lower Yuba and DC10, a considerable amount of morphologically identified EPT taxa were missed. This contrasts with what some other studies have shown where most (70%–95%) of the taxa morphologically identified were captured by the non-destructive metabarcoding of the ethanol preservative.

### 4.2 Challenges with viscous museum samples

Six samples from the California Department of Food and Agriculture (CDFA) presented challenges: these samples were notably darker, more viscous, and had a distinct sweet odor, which likely reflected differences in the preservative composition. Unlike the other ethanol samples, these did not evaporate properly during Speedvac processing and left considerable condensation behind. In an attempt to extract DNA from these samples, we applied a DNA precipitation method; however, this resulted in no recovery of arthropod taxa (protocol not shown).

We suspect that the chemical composition of the preservative in these particular CDFA samples was altered, possibly by additives or due to the samples themselves. The failure to extract DNA from these samples underscores the need for alternative approaches when processing ethanol from sources with unknown or varied preservative compositions. One possible explanation was that these samples and the ethanol preservative had a significant amount of propylene glycol (which can preserve DNA but to our knowledge has not been evaluated for preservation over time). Due to its widespread use in other applications and lack of flammability, this synthetic chemical can be used in a variety of invertebrate samples as an alternative to ethanol (Rubink et al. 2003; Martoni et al. 2021). We suspected that this chemical was added to the ethanol preservative of these malaise trap samples to reduce ethanol evaporation and further facilitate collection preservation.

### 4.3 DNA extraction

As in other studies, DNA extraction method was a major variable affecting metabarcoding results (Deiner et al. 2015). In our data, no extraction kit consistently outperformed the others for total arthropod detection or taxa validated by morphology. Prior work, however, has shown that soil kits sometimes could recover less invertebrate DNA, potentially due to degradation during bead-beating (Hermans et al. 2018; Martins et al. 2019).

Interestingly, PS improved fungal recovery in our samples, likely due to its optimized protocol for lysing tough fungal cells via bead-beating (Hermans et al. 2018). However due to the stochasticity observed in our extraction replicates, it is unclear whether our study had sufficient resolution to detect the kit-specific differences in arthropod recovery reported by others (Martins et al. 2019), but the improved fungal detection with PowerSoil was a clear and consistent pattern (Table S2).

A critical factor in this comparison was the nature of our samples. Unlike studies using clean ethanol with fresh specimens, our ethanol was ‘dirty,’ containing sediment, detritus, and organic debris from bulk BMI collections. This likely impacted DNA extraction and contributed to variability across replicates. While some studies have worked with similarly dirty ethanol (Martins et al. 2019; Zizka et al. 2019), many optimize for DNA recovery using clean ethanol (Hajibabaei et al. 2012; Carew et al. 2018; Chimeno et al. 2023). Future work should assess whether pretreatment, like filtering or sediment removal, could improve DNA yield and taxonomic recovery.

Even among contemporary BMI collections, storage conditions and sampling protocols varied. A key issue was inconsistent ethanol-to-material ratios. The LYR collection, for instance, had visibly low ethanol relative to biomass and debris. Other studies standardize this ratio for consistency (Martins et al. 2019), and future research should adopt this practice.

### 4.4 Lessons learned from sampling historic museum collection

Our results underscore several insights for research aiming to use the bycatch DNA for metabarcoding from historic invertebrate collection. First, arthropod recovery declined with sample age, consistent with DNA degradation over time (Raxworthy and Smith 2021). Though this pattern could be confounded by the collection method, as our oldest samples (>20 years) were exclusively pitfall traps from USGS Werc. Additionally, variation in the original sample diversity (site richness) could also contribute to recovery success. Targeting shorter *CO1* amplicons or multiplexing primer sets may improve amplification success and taxonomic recovery.

Storage conditions across museum collections were highly variable. Factors such as ethanol concentration, storage temperature, and ethanol to material ratio all likely influenced DNA integrity. For instance, samples stored in 70% ethanol (historically common) may suffer more severe degradation compared to those stored in 95% ethanol (Hajibabaei et al., 2012; Marquina et al. 2021). Some institutions also refrigerate samples, a practice that can slow degradation and preserve ethanol quality (Schiller et al. 2014).

Although accompanying metadata offered some insight into storage history, there were still many unknowns such as whether preservative fluids had been replaced or whether samples had been exposed to contaminants. These uncertainties represent a fundamental challenge when working with historical collections: preservation history is often incomplete or inconsistently documented, and even within a single collection, practices may vary widely.

A key methodological finding was that direct pipetting outperformed filtration, yielding higher arthropod richness from the same collections across Observed and Chao1 metrics. We hypothesize that pipetting may better retain intracellular DNA or coarse tissue fragments suspended in the ethanol. However, we did not observe significant differences in community composition or evenness metrics (Shannon, Simpson), indicating that while pipetting recovers more taxa, the overall community composition remains similar. Based on this we recommend museums use the pipetting method.

Contamination posed another persistent challenge. Several USGS WERC samples, for example, produced *CO1* PCR bands but sequencing revealed only human DNA reads and no arthropod taxa. This underscores the importance of rigorous contamination control and, where feasible, the use of human DNA blocking primers when working with museum collections.

Our successful recovery of fungal DNA from all 70 museum samples, including collections up to 26 years old demonstrates that non-destructive metabarcoding of the preservative fluid can capture more than just invertebrate diversity but potentially the microbial community associated with the specimens themselves. The recovery of fungal taxa that are known to be insect associates such as pathogens and symbionts confirms that biologically meaningful associations can persist alongside the inevitable storage contaminants. The interpretive challenges lie in distinguishing the original specimen-associated fungi from post collection colonizers. However this challenge does not diminish the value of fungal metabarcoding from historic insect collections. As methods improve and reference databases continue to expand, metabarcoding will only get more powerful, and the preserved DNA can be reanalyzed with better resolution. As museums increasingly recognize their role in a rapidly changing climate, our results demonstrate the potential of metabarcoding to not only characterize the specimens contained within collections, but understanding how host-microbial interactions shift over time.

### 4.5 Integrating molecular and morphological data is appealing for future stream surveys

Our comparisons highlight the importance of exercising caution when interpreting taxonomic assignments from eDNA data. We started with framework 1 with no filtering enabling a broad comparison of taxonomic assignments. However, errors in reference databases, such as sequences associated with misidentified specimens or the proliferation of provisional accession numbers, can artificially inflate richness estimates in the eDNA data. For example, when looking at species assignments from DC10 and the LYR generated from molecular methods, we saw a proliferation of BOLD accession numbers for the family Chironomidae. These assignments reflect amplicon sequence variation rather than necessarily distinct biological species as slight sequence variation or a few nucleotide mismatches within the same species may lead to multiple unique taxon assignments. It is unclear whether species assignments of amplicon sequence variation represent distinct biological species or whether small sequence variations within the same species can lead to multiple unique taxon assignments. If the latter is true, species with high intraspecific variation could artificially inflate species diversity estimates generated from metabarcodes. Other DNA artifacts such as Nuclear embedded mitochondrial DNA—NUMTs, can inflate richness in bulk metabarcoding (Hebert et al. 2023). Conversely, the absence of DNA barcodes for certain taxa can lead to an underestimation of true diversity, particularly in groups with high cryptic diversity. These limitations emphasize the need for more comprehensive DNA barcoding efforts and routine curation of NCBI data to improve records.

Despite these limitations, eDNA provides insights into taxa overlooked by morphology and highlights knowledge gaps. Its putative species estimates guide future work by identifying taxa for re-examination and areas needing better reference coverage. Combining metabarcoding with morphological identifications improves both biodiversity estimates and taxonomic resolution.

Framework 1 allowed richness quantification, while framework 2 aligned eDNA data with bioassessment frameworks like SAFIT Level Two, essential for regulatory monitoring. This standardization enables direct comparisons with morpho-ID data and evaluates how well eDNA complements traditional methods. Framework 2 revealed that while eDNA missed morpho-identified taxa, it consistently detected additional lineages, often resolving shared taxa to higher levels. This demonstrates the promise of eDNA while grounding it within the expectations of traditional BMI monitoring.

An important distinction lies in the spatial scale of biodiversity captured. Water eDNA consistently recovered more taxa than ethanol metabarcoding and morpho-IDs because water integrates DNA from the entire upstream catchment, acting as a composite biodiversity signal (Deiner et al. 2016). This makes water eDNA ideal for broad biodiversity surveys but less suited for site-specific assessments. Ethanol metabarcoding, derived from localized bulk samples, provides a finer spatial resolution comparable to morpho-IDs.

Our results show that metabarcoding captures additional biodiversity, including freshwater sponges and jellyfish, typically overlooked by kicknet surveys. Cryptic taxa like oligochaetes were also resolved to finer taxonomic levels with eDNA. However, read abundance did not reliably reflect organismal abundance due to variability in DNA shedding, biomass, PCR bias, and sequencing artifacts. This reinforces that presence/absence data, rather than abundance estimates, should be used when integrating molecular and morphological datasets in agreement with Brasseur et al. (2023).

In summary, DNA metabarcoding can improve taxonomic resolution, detect cryptic taxa, and reveal upstream biodiversity signals missed by local sampling. It should be used to complement, not replace, expert morphological identification. Indeed, morphological verifications will also improve the resolution and accuracy of reference databases of *CO1* sequences. Integrating both provides the most complete picture of stream biodiversity.

## Supplementary Material

Supplemental_Figure_1_esag001

CIB_Manuscript_Supplemental_Table1_(1)_esag001

CIB_Manuscript_Supplemental_Table2_(2)_esag001

CIB_manuscript_Supplemental_Table3_(2)_esag001

CIB_Manuscript_Supplemental_Table4_(1)_esag001
