# Supplementary material for: Bycatch in a bottle: what taxa are recoverable from metabarcoding DNA in historical invertebrate collection preservative fluid?
Source: J Hered. 2026 Jan 12;117(5):1036–48. doi: 10.1093/jhered/esag001 (PMC13539438; doi:10.1093/jhered/esag001)
Supplement: Supplementary file 1 [file supplemental_figure_1_esag001.pdf]

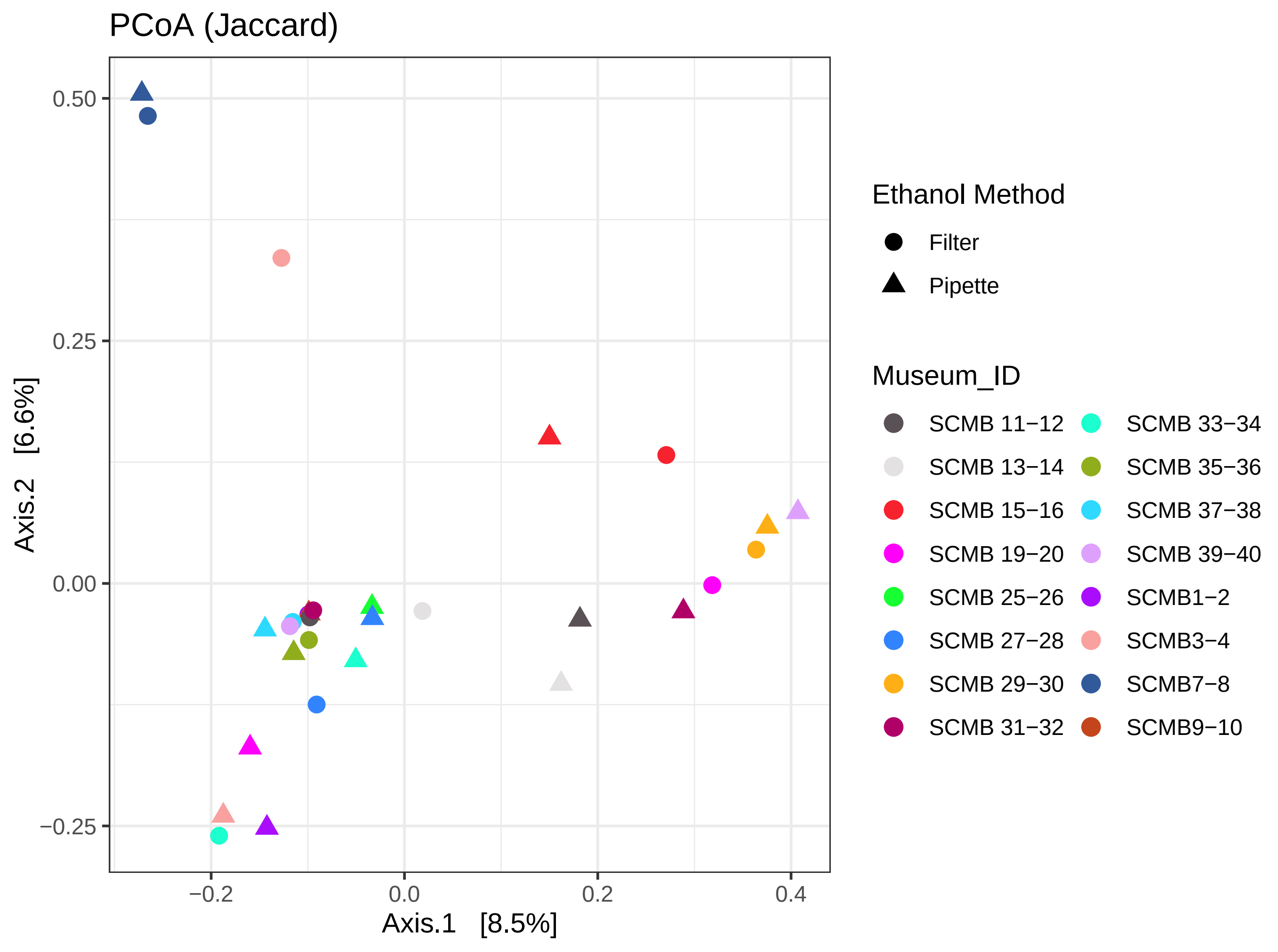

PCoA (Jaccard)
0.50
0.25
0.00
−0.25
Axis.2 [6.6%]
−0.2
0.0
0.2
0.4
Axis.1 [8.5%]
Ethanol Method
Filter
Pipette
Museum_ID
SCMB 11−12
SCMB 13−14
SCMB 15−16
SCMB 19−20
SCMB 25−26
SCMB 27−28
SCMB 29−30
SCMB 31−32
SCMB 33−34
SCMB 35−36
SCMB 37−38
SCMB 39−40
SCMB1−2
SCMB3−4
SCMB7−8
SCMB9−10
